# Comparative Analysis of Machine-Learning Algorithms for Accurate Diagnosis of Lung Diseases Using Chest X-ray Images: A Study on Balanced and Unbalanced Data on Segmented and Unsegmented Images

**DOI:** 10.7759/cureus.53282

**Published:** 2024-01-31

**Authors:** Sreedevi Jasthy, Krishnamurthy Ramasubramanian, Radhakrishna Vangipuram, Satyanarayana Bollu

**Affiliations:** 1 Department of Computer Science and Engineering, Koneru Lakshmaiah Education Foundation, Mahatma Gandhi Institute of Technology, Hyderabad, IND; 2 Department of Computer Science and Engineering, Koneru Lakshmaiah Education Foundation, Hyderabad, IND; 3 Department of Information Technology, Vallurupalli Nageswara Rao Vignana Jyothi Institute of Engineering and Technology, Hyderabad, IND; 4 Department of Mechanical Engineering, Vallurupalli Nageswara Rao Vignana Jyothi Institute of Engineering and Technology, Hyderabad, IND

**Keywords:** balanced dataset, attribute reduction, machine learning, cxr, lung diseases

## Abstract

The study focused on the accurate diagnosis of lung diseases, considering the high number of lung disease-related deaths in the world. Chest x-ray images were used as they are a cost-effective and widely available diagnostic tool. Eight different machine learning algorithms were evaluated: Logistic Regression, Naive Bayes, k-Nearest Neighbors (kNN), Decision Tree, Random Forest, Support Vector Machine (SVM), Ridge, and Least Absolute Shrinkage and Selection Operator (LASSO). The study evaluated balanced and imbalanced datasets and looked at both segmented and unsegmented chest x-ray images. COVID-19, pneumonia, normal, and others were the four classes that were used in the investigation. Prior to attribute reduction, Decision Tree and Random Forest performed well on the balanced dataset, obtaining 74% test accuracy and 92% training accuracy. SVM functioned well as well, obtaining a 74% test accuracy. Principal Component Analysis (PCA) and Linear Discriminant Analysis (LDA) are two attribute reduction approaches that were applied. Decision Trees and Random Forests were able to attain the maximum training accuracy of 92%, while SVM was able to retain a test accuracy of 74% after attribute reduction. The findings also imply that some algorithms' performance may be enhanced by attribute reduction methods like PCA and LDA. For imbalanced data, Random Forest and SVM perform the best in terms of balanced accuracy of 80%. However, further research and experimentation may be needed to optimize the models and explore other potential algorithms or techniques.

## Introduction

Careful thought and the right approaches are needed to address the issues of high non-linearity of medical images and imbalanced datasets. This raises the requirement for effective machine learning models [1,2] for illness diagnosis, especially in the setting of binary and multi-class classification. The following recommendations may help to increase detection rates, precision, and accuracy.

Class Balancing Techniques like sampling the majority class or oversampling the minority class such as the synthetic minority over-sampling technique (SMOTE) can help balance the class distribution and lessen the effects of imbalanced datasets.

The best-performing machine learning model can be found by utilizing rigorous cross-validation [3] procedures and suitable evaluation metrics such as accuracy, recall, F1 score, receiver operating characteristic (ROC), and area under curve (AUC). By utilizing feature extraction methods like PCA and LDA, it is possible to extract high-level features from medical pictures while overcoming the non-linearity problem. To improve accuracy and precision rates, certain medical imaging datasets can benefit from fine-tuning or transfer learning.

Ensemble methods improve performance by combining many machine learning models. By utilizing the advantages of several models, strategies like bagging (e.g., Random Forest) or boosting (e.g., AdaBoost, Gradient Boosting) can increase accuracy, precision, and detection rates. Hyperparameter tuning helps to achieve better performance by optimizing the hyperparameters of machine learning models through methods such as grid search or Bayesian optimization.

Finding the ideal balance between precision and accuracy is critical, depending on the particulars of the disease diagnosis issue. Working with domain experts, such as radiologists or other medical professionals, can help construct more precise and accurate machine-learning models by offering insightful information about the properties of medical pictures. It is significant to note that the efficacy of these methods may differ based on the datasets.

In the context of medical image analysis, experimentation, iterative refinement, and ongoing assessment are essential for creating an effective machine-learning model. The aim of this study is to compare and analyze the performance of different machine learning algorithms on balanced and imbalanced data for diagnosing different lung diseases using chest x-ray images.

## Technical report

Problem statement

The goal of the study is to use machine learning algorithms to correctly diagnose different lung diseases from chest x-ray images [4-6]. Utilizing machine learning models can lessen the workload for physicians while producing outcomes that are more precise. Evaluating and contrasting the performance of several machine learning algorithms is probably part of the study to find out which algorithm produces superior results. Data gathering, preprocessing, feature extraction, algorithmic selection, model training and evaluation, validation, and comparative analysis are some of the steps that may be involved in this process. The implementation of this process yields significant knowledge regarding the optimal machine learning model for multiple lung disease detection from chest x-ray images. The findings can direct the creation of a reliable and accurate model that will help medical practitioners diagnose lung disorders more accurately.

System architecture

**Figure 1:**
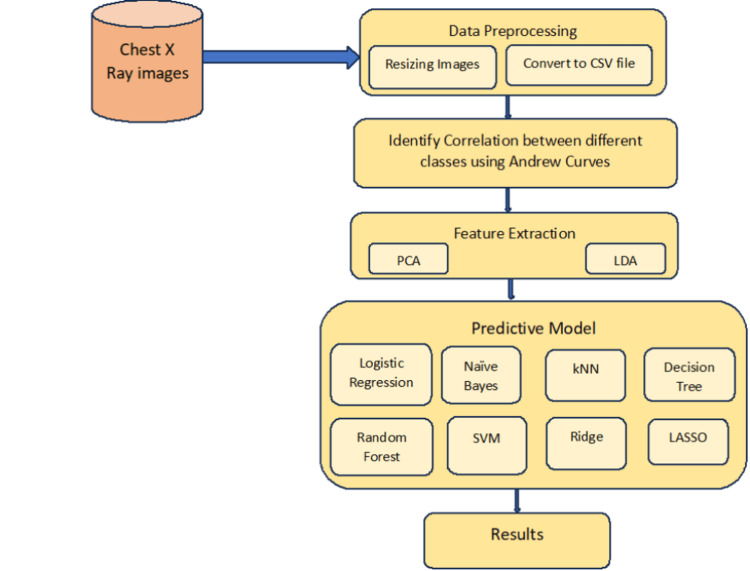
System architecture before segmentation

Figure 1 describes the system architecture of the workflow before image segmentation. The dataset is collected from different repositories like IEEE, Mendel, and Kaggle. The dataset consists of images categorized into different classes such as COVID-19, pneumonia, normal and other. The gathered images are scaled to a standard size. Then the images are converted into Comma Separated Value (CSV) files. The process of converting csv file includes loading the original image, converting it to greyscale and further converting the greyscale image to the array. This array is then written to a CSV file using the ‘csv’ library of Python. This step ensures that the images are ready for processing in a consistent format. The correlation between the images is found using Andrew curves. Andrew curves offer a visual aid for locating regions where variables group together and exhibit correlation. Understanding the connections between the various aspects or attributes in the dataset is made easier by this approach. There are training and test datasets within the dataset. This section enables testing the models' performance using untested data from the test dataset and training the models on the training dataset. Training datasets are used to create predictive models. The models are trained using a variety of machine learning algorithms, and they are assessed using performance indicators. The metrics offer information about the models' specificity, sensitivity, recall, accuracy, precision, and F1 score. Techniques like principal component analysis (PCA) and linear discriminant analysis (LDA) are used to lower the dataset's dimensionality. While LDA looks for the discriminant features that best divide the classes, PCA seeks to identify the most significant variances in the data. The predictive models are trained once more using the condensed attribute sets that are produced by PCA and LDA. The impact of attribute reduction on the models' accuracy, precision, recall, F1-score, specificity, and sensitivity is measured using the same metrics as previously. The study is to examine the performance of several predictive models, specifically using PCA and LDA [7], before and after attribute reduction by employing this approach. This allows for a comprehensive evaluation of the models' performance and the effectiveness of attribute reduction techniques in the context of lung disease detection from chest x-ray images.

**Figure 2:**
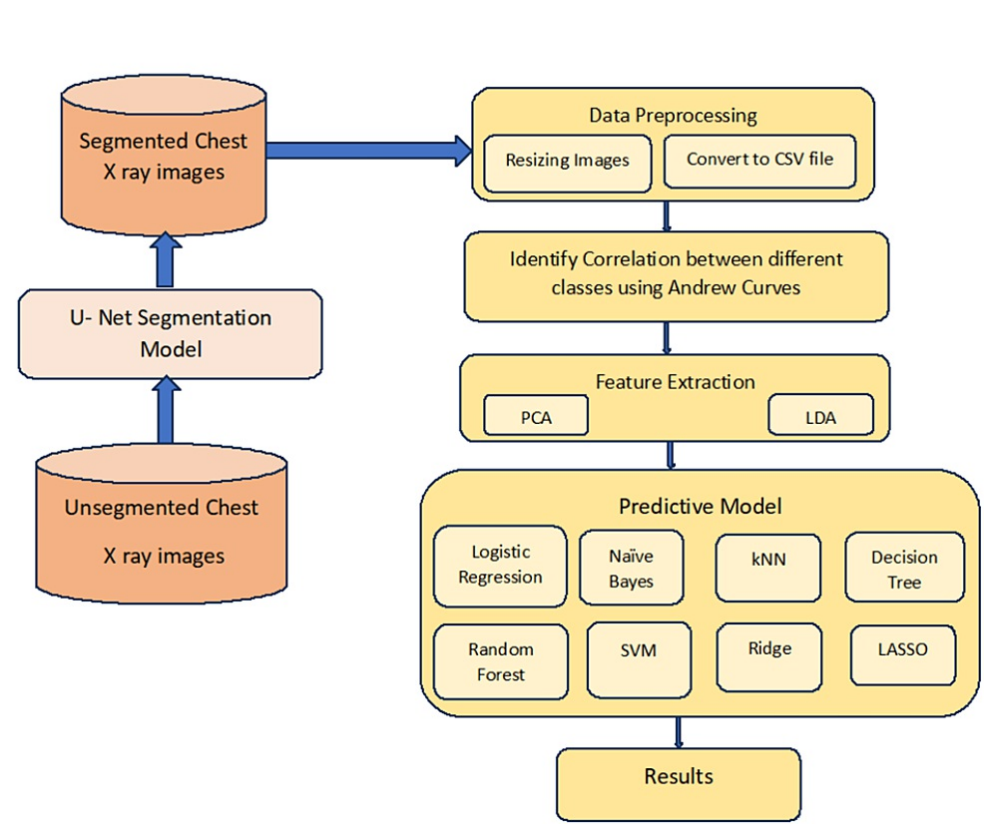
System architecture-segmented images

The ordinary x-ray images are segmented using a CNN model [8-14]. Then the segmented images are resized and converted to CSV format with 784 attributes. The same process is followed for the segmented data as was for unsegmented data. Figure 2 is the architecture of segmented images.

Initially, the study focuses on visualizing the relationship between the classes of the dataset using the Andrew plot. The Andrews plot works by using a curve created by evaluating a finite Fourier series to represent each observation. By breaking down a function into a sum of sinusoidal components, the Fourier series makes it possible to express complex curves with simpler trigonometric functions. With the y-axis indicating the value of the Fourier series at each point and the x-axis representing the evaluation points along the curve, the Andrews plot shows every curve in a 2D graphic. By examining the patterns and clusters formed by the curves, we can identify correlations and similarities among the different variables for different classes. Areas where variables correlate closely have similar curve shapes, while areas with little correlation or outliers will exhibit a distinct and different curve shapes.

Analysis (Balanced Dataset)

The balanced dataset contains an equal number of samples for all the classes. In this section, the classifiers are evaluated before attribute reduction and after attribute reduction for the balanced dataset, based on their accuracy in correctly classifying instances, true positive rate (TPR), false positive rate (FPR), precision, recall, and F-measure.

**Figure 3:**
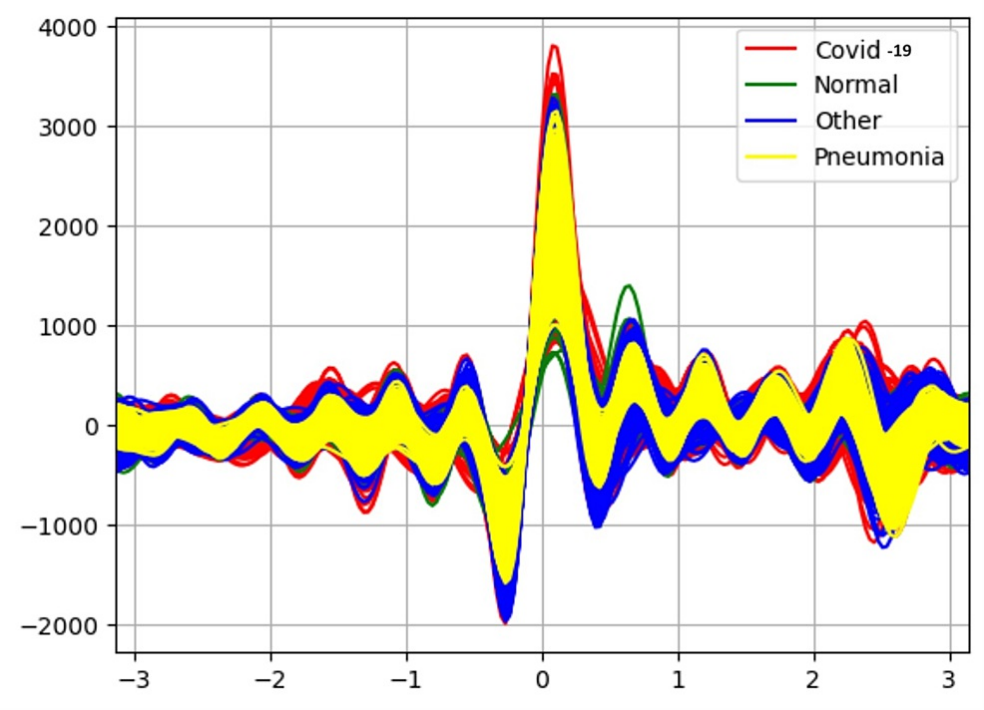
Andrew-curve-without applying attribute selector

Results in Table 1 indicate the performance of each classifier without the application of an attribute selector. The Random Forest classifier is able to classify the data with 90% accuracy. Figure 3 is an Andrew curve without applying an attribute selector representing the non-linearity between different classes. These curves are obtained using pandas “plotting.andrews_curves.” They are then plotted using Matplotlib.

**Table 1:** Without applying attribute selector TPR - True Positive Rate, FPR - False Positive Rate

Classifier	Correctly classified	Incorrectly classified	TPR	FPR	Precision	Recall	F-Measure
NAÏVE BAYES	48.63	51.36	0.48	0.16	0.47	0.48	0.45
LOGISTIC	62.09	37.90	0.62	0.12	0.61	0.62	0.61
J48	82.26	17.73	0.82	0.05	0.82	0.82	0.82
RANDOMFOREST	90.86	9.13	0.90	0.03	0.91	0.90	0.90

**Figure 4:**
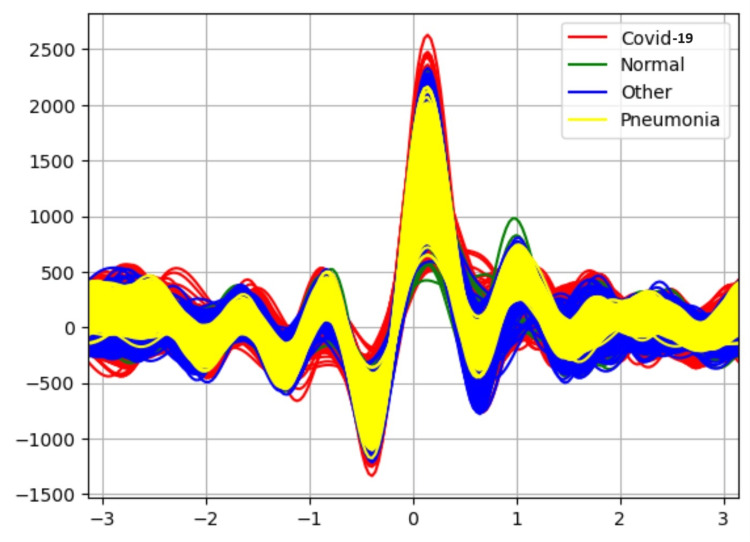
Andrew-curve-after applying attribute selector-CFS-BESTFIRST

Table 2 results indicate the performance of each classifier after applying the attribute selector CFS with the BESTFIRST search. The Random Forest classifier is able to classify the data with 90% accuracy. Figure 4 shows the Andrew curve after applying attribute selector-CFS-BESTFIRST. This Andrew curve shows better co-relation between different classes than before applying the attribute selector.

**Table 2:** Applying attribute selector-CFS - BESTFIRST CFS - Correlation-based feature selection

Classifier	Correctly classified in Percentage	Incorrectly classified in Percentage	TPR	FPR	PRESICION	RECALL	F-MEASURE
NAÏVE BAYES	48.55	51.44	0.48	0.16	0.47	0.48	0.45
LOGISTIC	58.13	41.86	0.58	0.14	0.57	0.58	0.57
J48	81.40	18.55	0.81	0.06	0.81	0.81	0.81
RANDOMFOREST	90.81	9.18	0.90	0.03	0.90	0.90	0.90

**Figure 5:**
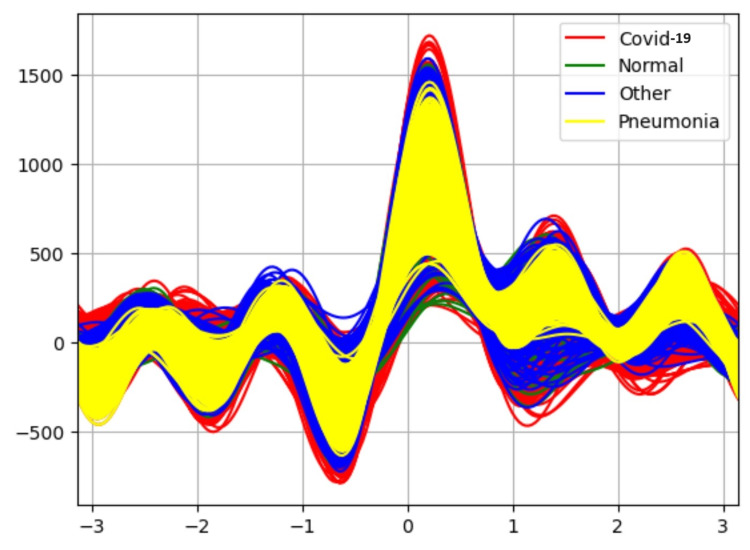
Andrew-curve-attribute selector-INFOGAIN-RANKER

Table 3 results represent the performance of each classifier after applying the INFOGAIN-RANKER attribute selector. The Random Forest classifier is able to classify the data with 90% accuracy and the performance of other classifiers is also increased slightly. Figure 5 shows Andrew's curve for the attribute selector Infogain-RANKER. This Andrew curve shows a better co-relation between pneumonia and different classes.

**Table 3:** Attribute selector-INFOGAIN-RANKER

Classifier	Correctly classified	Incorrectly classified	TPR	FPR	PRESICION	RECALL	F-MEASURE
NAÏVE BAYES	48.63	51.36	0.48	0.18	0.47	0.48	0.45
LOGISTIC	62.09	37.90	0.62	0.12	0.61	0.62	0.61
J48	82.264	17.73	0.82	0.05	0.82	0.82	0.82
RANDOMFOREST	90.86	9.13	0.90	0.03	0.91	0.90	0.90

**Figure 6:**
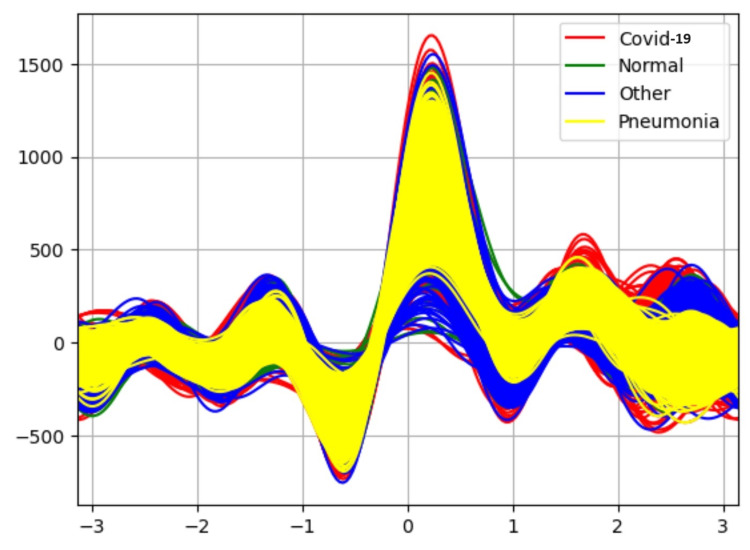
Andrew-curve-attribute selector-PCA-RANKER

Table 4 results represent the performance of each classifier after applying the PCA-RANKER attribute selector. The PCA RANKER attribute selector uses PCA to reduce the dimensionality of the dataset, potentially improving the performance of the Naive Bayes classifier but decreasing the performance of J48 and Logistic regression. Figure 6 shows Andrew's curve for the attribute selector PCA-RANKER. The non-linearity between different classes is reduced slightly. 

**Table 4:** Attribute selector-PCA-RANKER PCA - Principle component analysis

Classifier	Correctly classified	Incorrectly classified	TPR	FPR	PRESICION	RECALL	F-MEASURE
NAÏVE BAYES	56.2	43.79	0.56	0.14	0.51	0.56	0.55
LOGISTIC	55.7	44.28	0.55	0.14	0.55	0.55	0.55
J48	80.35	19.64	0.80	0.06	0.80	0.80	0.80
RANDOMFOREST	90.86	9.134	0.90	0.03	0.91	0.90	0.90

Analysis (Unbalanced Dataset)

In this section, an unbalanced dataset is considered for evaluating the performance of different classifiers before attribute reduction and after attribute reduction. Table 5 displays the performance of different classifiers before applying attribute selector and after applying attribute selectors like CFS, infogain, and PCA. It shows that, not much difference between before and after attribute selectors. However, the performance of the classifiers is better for a balanced dataset than an unbalanced dataset.

**Table 5:** Analysis of unbalanced dataset

Without Applying Attribute selector							
Classifier	Correctly classified	Incorrectly classified	TPR	FPR	PRESICION	RECALL	F-MEASURE
NAÏVE BAYES	43.83	56.16	0.43	0.16	0.46	0.43	0.42
LOGISTIC	58.91	41.08	0.58	0.15	0.58	0.58	0.58
J48	58.85	41.14	0.58	0.15	0.58	0.58	0.58
RANDOMFOREST	64.66	35.33	0.64	0.12	0.64	0.64	0.64
Applying Attribute selector - CFS							
NAÏVE BAYES	44.31	55.68	0.44	0.16	0.47	0.44	0.42
LOGISTIC	55.18	44.81	0.55	0.17	0.55	0.55	0.55
J48	58.47	41.52	0.58	0.15	0.58	0.58	0.58
RANDOMFOREST	63.28	36.71	0.63	0.13	0.63	0.63	0.63
Applying Attribute selector - infogain							
NAÏVE BAYES	43.83	56.12	0.43	0.16	0.14	0.43	0.42
LOGISTIC	58.91	41.08	0.58	0.15	0.58	0.58	0.58
J48	58.85	41.14	0.58	0.15	0.58	0.58	0.58
RANDOMFOREST	64.6	35.38	0.64	0.12	0.64	0.64	0.64
Applying Attribute selector - PCA							
NAÏVE BAYES	52.47	47.5	0.52	0.15	0.55	0.52	0.52
LOGISTIC	51.41	48.58	0.51	0.18	0.51	0.51	0.51
J48	58.43	41.56	0.58	0.15	0.58	0.58	0.58
RANDOMFOREST	62.23	37.76	0.62	0.13	0.62	0.62	0.62

**Figure 7:**
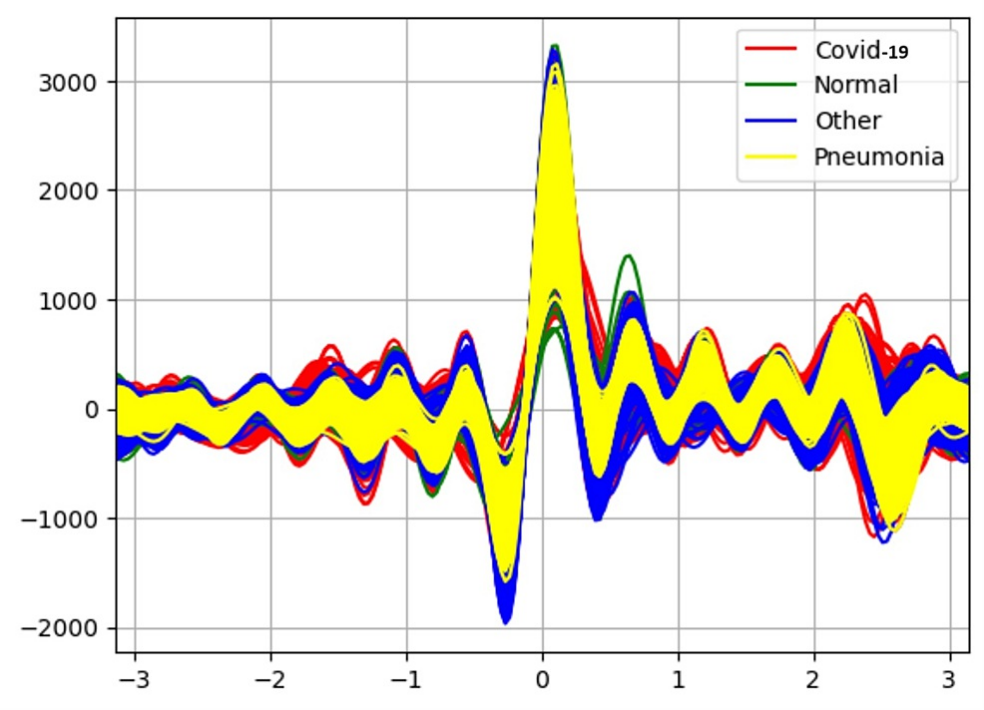
Andrew curve without applying attribute selector for unbalanced dataset

Figure 7 represents higher nonlinearity before applying attribute selector methods for unbalanced data.

**Figure 8:**
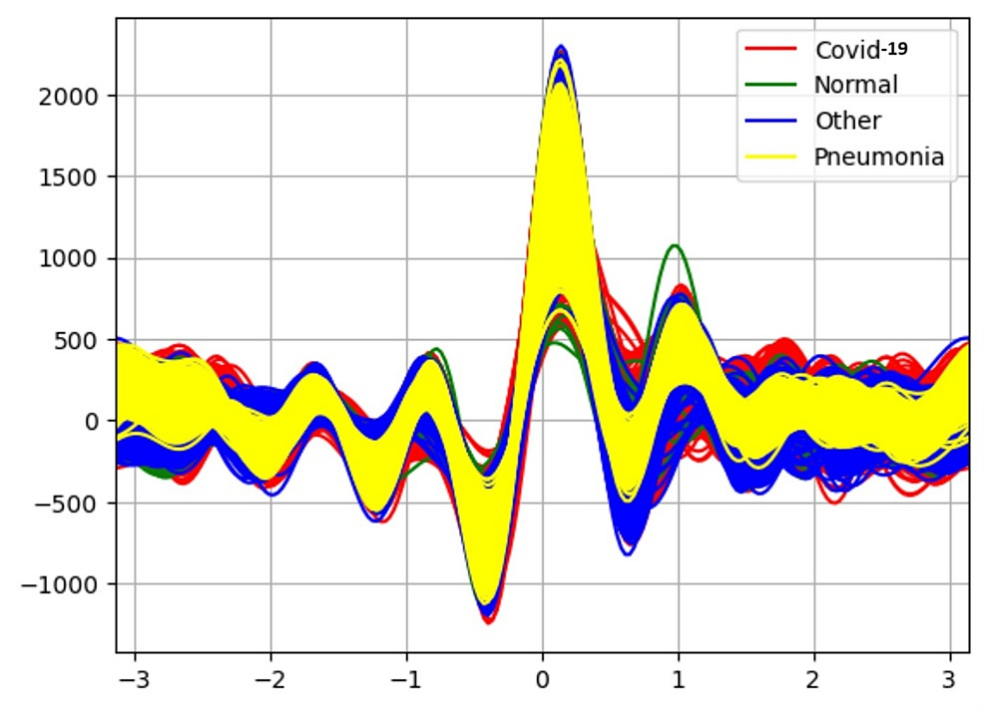
Andrew curve applying attribute selector-CFS for unbalanced dataset

**Figure 9:**
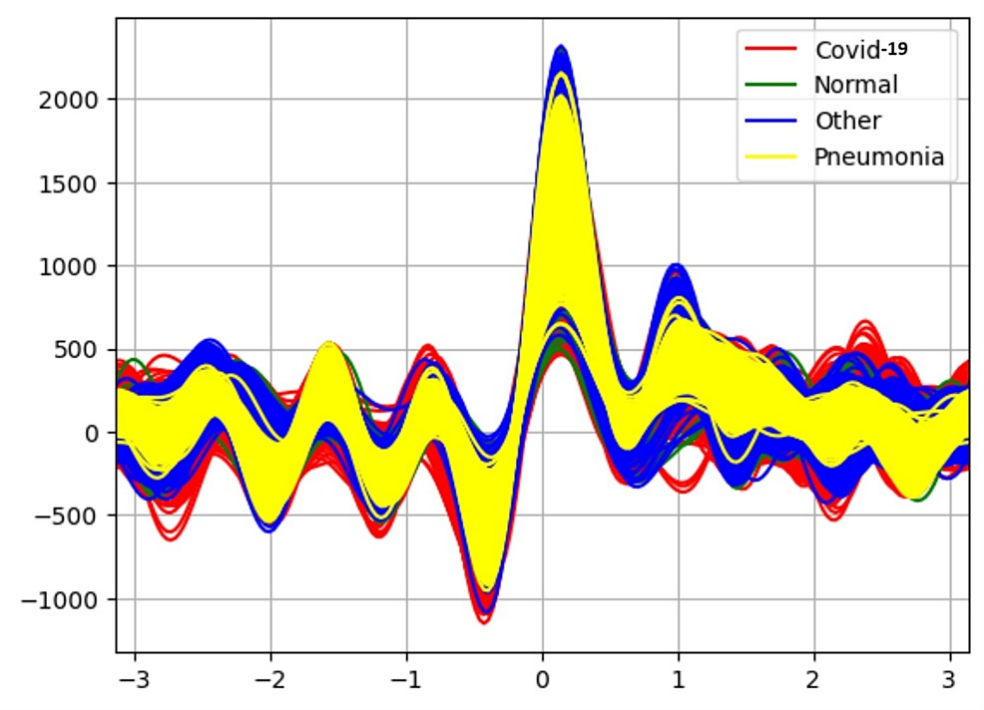
Andrew curve applying attribute selector-infogain for unbalanced dataset

**Figure 10:**
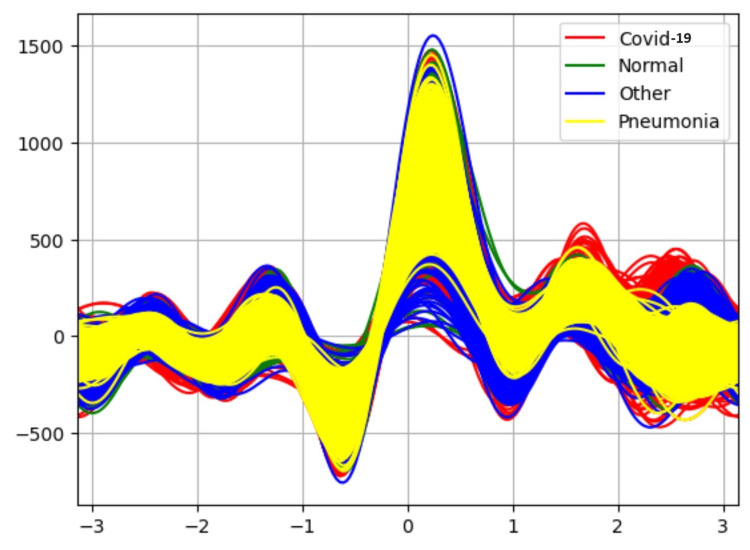
Andrew curve applying attribute selector-PCA for unbalanced dataset

In the case of the CFS attribute selector from Figure 8, it seems that reducing linearity may have negatively impacted the performance of the classification algorithms. This could be due to the removal of features that were informative for classification, leading to a loss of important information. For the Infogain attribute selector from Figure 9, the slight reduction in nonlinearity without significant changes in performance suggests that the selected attributes may have provided some additional discriminatory power, but not to a significant extent. On the other hand, from Figure 10, it is observed that the PCA algorithm has shown a slight reduction of nonlinearity between different classes. PCA reduces the dimensionality of the dataset while retaining as much information as possible. This dimensionality reduction can help alleviate the curse of dimensionality and improve the efficiency and performance of the classifiers. The Naive Bayes classifier has improved its performance slightly than the remaining attribute selection methods CFS and Infogain.

Dataset

**Figure 11:**
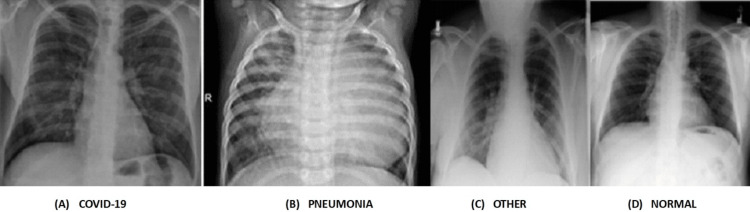
(A-D) Chest x-ray images collected from Kaggle dataset

**Figure 12:**
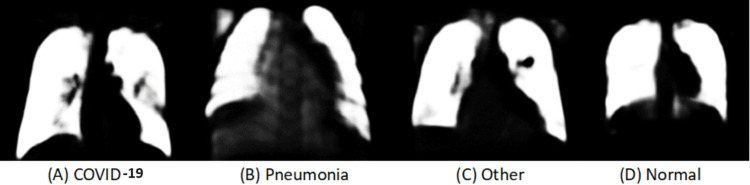
(A-D) Segmented chest x-ray images

The dataset used in this study is a combination of various open-source datasets like the IEEE repository, Mendel repository, and Kaggle. The normal lung images are from the COVID-19 Radiography Database. Different types of Pneumonia images are from the pneumonia x-ray images dataset. The other lung disease images are from the NIH Chest X-rays database and COVID-19 images are from IEEE, Mendel, and Kaggle repositories. Total of 12,574 images are used in this study of which 10,059 are used for training and 2,515 are used for testing. All the 12,574 ordinary images are segmented using a model built on convolutional neural network (CNN) using U-Net Architecture. Figures 11A-11D show the sample images of different classes and Figures 12A-12D show the segmented images of different classes.

Training

The model is trained and built using various Machine Learning algorithms. The different algorithms used are Logistic Regression, Naïve Bayes, k-nearest Neighbors (kNN), Decision Tree, Random Forest, Support Vector Machine, Ridge, and least absolute shrinkage and selection operator (LASSO). When the dimensionality of pre-processed data was reduced using PCA, it gave a total of 37 components for unsegmented data and 117 for segmented data. Finally, the important features are extracted and used to train the model. Using LDA on the pre-processed data for dimensionality reduction, three components were extracted. The data after applying LDA are used to train different machine-learning models.

## Discussion

Unsegmented images (balanced dataset)

Table 6 shows the evaluation metrics for several classification models before attribute reduction and after attribute reduction for unsegmented images. The attribute reduction methods used include LDA and PCA. The machine learning models used in the study include Logistic Regression, Naive Bayes, kNN, Decision Tree, Random Forest, Support Vector Machine (SVM), ridge regression, and LASSO regression.

**Table 6:** Metrics of different classifiers for Unsegmented images Before and After attribute Reduction

Classification	Precision	Recall	F-1 Score	Specificity	ROC	Training Accuracy	Testing Accuracy
Logistic Regression	0.64	0.64	0.64	0.88	0.76	72.5	64.55
Naive Bayes	0.54	0.54	0.51	0.84	0.69	52.3	54.29
kNN (k=3)	0.66	0.66	0.66	0.88	0.77	81.6	66.46
Decision Tree	0.54	0.54	0.54	0.84	0.69	92.97	54.09
Random Forest	0.67	0.67	0.66	0.88	0.78	92.97	67.02
Support Vector Machine	0.74	0.74	0.74	0.91	0.83	81.29	74.66
ridge	0.64	0.64	0.64	0.88	0.76	72.78	64.24
LASSO	0.66	0.66	0.65	0.88	0.77	73.88	65.51
LDA - Attribute Reduction							
Logistic Regression	0.64	0.64	0.64	0.88	0.76	72.27	64.39
Naive Bayes	0.63	0.63	0.63	0.87	0.75	72.30	63.52
kNN (k=3)	0.58	0.58	0.58	0.85	0.72	80.04	58.19
Decision Tree	0.53	0.529	0.52	0.84	0.68	92.97	52.90
Random Forest	0.58	0.58	0.58	0.86	0.72	92.97	58.59
Support Vector Machine	0.65	0.65	0.64	0.88	0.77	73.11	65.19
ridge	0.63	0.64	0.63	0.87	0.76	72.81	64.04
LASSO	0.64	0.64	0.64	0.88	0.76	72.76	64.24
PCA - Attribute Reduction							
Logistic Regression	0.63	0.63	0.63	0.87	0.76	64.35	63.68
Naive Bayes	0.59	0.59	0.58	0.86	0.73	58.86	59.94
kNN (k=3)	0.66	0.66	0.66	0.87	0.83	82.03	66.07
Decision Tree	0.52	0.52	0.52	0.83	0.68	92.97	52.30
Random Forest	0.65	0.65	0.64	0.88	0.77	92.97	65.07
Support Vector Machine	0.74	0.74	0.73	0.913	0.82	78.54	74.02
ridge	0.64	0.64	0.64	0.88	0.76	64.30	64.59
LASSO	0.65	0.65	0.64	0.882	0.76	64.98	64.75

The best results for the training dataset of unsegmented images, before attribute reduction and after attribute reduction, are observed for Decision Tree and Random Forest, both achieving a training accuracy of 92.9%. SVM achieves the best result prior to attribute reduction for the test dataset of unsegmented pictures, with an accuracy of 74%. The SVM's testing accuracy drops to 65.1% when attribute reduction using LDA is applied. In a similar vein, the SVM attains a 74% testing accuracy after utilizing PCA for attribute reduction. Remember that in the balanced dataset, accuracy is one measure to consider, to fully comprehend the model's performance. It's critical to assess additional metrics like precision, recall, F1 score, and specificity. The Support Vector Machine model outperformed all other models before attribute reduction in terms of precision, recall, F1 score, specificity, and ROC for the given dataset.

Segmented images (balanced dataset)

From Table 7, the findings give a general picture of each model's accuracy prior to attribute reduction. Both Random Forest and Decision Tree achieve 100% training accuracy, showing the greatest results for the training dataset of unsegmented images, both before and after attribute reduction. Random Forest achieves the best performance prior to attribute reduction for the test dataset of unsegmented pictures, with an accuracy of 96%. After applying PCA and LDA for attribute reduction, the Decision Tree and Random Forest training accuracy is 100%. It is found that the Random Forest model achieved the highest testing accuracy of 96% before attribute reduction. However, after applying attribute reduction using LDA and PCA, the accuracy of the models decreased and did not show improved performance compared to the original models. But it is important to note that the accuracy is not the only measure to identify the model performance, but other metrics also play important role in medical images.

**Table 7:** Metrics of segmented images for balanced dataset

After Segmentation							
Before Attribute Reduction							
Classification	Precision	Recall	F-1 Score	Specificity	ROC	Training Accuracy	Testing Accuracy
Logistic Regression	0.83	0.83	0.83	0.94	0.88	90.67	83.14
Naive Bayes	0.61	0.48	0.44	0.82	0.64	49.37	48.82
kNN (k=3)	0.74	0.74	0.74	0.91	0.82	86.74	74.03
Decision Tree	0.91	0.91	0.91	0.90	0.97	100	91.01
Random Forest	0.96	0.96	0.96	0.98	0.97	100	96.93
Support Vector Machine	0.84	0.84	0.84	0.94	0.89	92.26	84.73
ridge	0.75	0.75	0.75	0.917	0.83	81.12	75.38
LASSO	0.86	0.86	0.86	0.95	0.90	91.06	85.92
LDA - Attribute Reduction							
Logistic Regression	0.75	0.75	0.75	0.91	0.83	81.42	75.18
Naive Bayes	0.75	0.74	0.74	0.91	0.82	80.84	74.43
kNN (k=3)	0.72	0.72	0.72	0.90	0.81	86.55	72.36
Decision Tree	0.69	0.69	0.69	0.89	0.79	100	69.38
Random Forest	0.74	0.74	0.74	0.91	0.83	100	74.91
Support Vector Machine	0.75	0.75	0.75	0.91	0.83	82.08	75.58
ridge	0.75	0.75	0.75	0.91	0.83	81.16	75.50
LASSO	0.75	0.75	0.75	0.91	0.83	81.57	75.18
PCA - Attribute Reduction							
Logistic Regression	0.79	0.79	0.79	0.92	0.86	80.95	79.04
Naive Bayes	0.51	0.40	0.37	0.80	0.61	41.11	40.95
kNN (k=3)	0.74	0.74	0.74	0.91	0.82	86.99	74.03
Decision Tree	0.62	0.62	0.62	0.87	0.74	100	62.02
Random Forest	0.75	0.75	0.75	0.91	0.83	100	75.82
Support Vector Machine	0.84	0.83	0.83	0.94	0.89	90.16	83.93
ridge	0.71	0.71	0.71	0.90	0.80	72.97	71.21
LASSO	0.78	0.78	0.78	0.92	0.85	79.46	77.85

Stratified k-fold for unbalanced data

Table 8 shows the unbalanced data results after applying stratified k-fold cross validation technique. When dealing with unbalanced data, it is important to consider evaluation metrics that are robust to class imbalance. One commonly used metric is the balanced accuracy which is the arithmetic mean of sensitivity and specificity. The recall metric is also used to assess the performance of the models. Based on the balanced accuracy metric, which considers both sensitivity and specificity, the models can be ranked for testing accuracy as follows:

1. Random Forest (Test Balanced Accuracy: 0.8) in fold 3

2. SVM (Test Balanced Accuracy: 0.8) in fold 3

3. k-NN (Test Balanced Accuracy: 0.77) in fold 3

4. LASSO (Test Balanced Accuracy: 0.76) in fold 10

5. Logistic Regression (Test Balanced Accuracy: 0.75) in fold 10

6. Ridge (Test Balanced Accuracy: 0.73) in fold 6

7. Decision Tree (Test Balanced Accuracy: 0.72) an in fold 8

8. Naive Bayes (Test Balanced Accuracy: 0.69) in fold 6

**Table 8:** Stratified k-fold for unbalanced data

Logistic Regression				NAIVE BAYES			
FOLD	METRICS	TRAINING	TEST	FOLD	METRICS	TRAINING	TEST
F10	RECALL	0.65	0.62	F6	RECALL	0.56	0.55
	SPECIFICITY	0.89	0.88		SPECIFICITY	0.84	0.83
	PRECISION	0.65	0.62		PRECISION	0.53	0.52
	BALANCED ACCURACY	0.77	0.75		BALANCED ACCURACY	0.7	0.69
	F1-SCORE	0.68	0.67		F1-SCORE	0.48	0.45
kNN				DECISION TREE			
FOLD	METRICS	TRAINING	TEST	FOLD	METRICS	TRAINING	TEST
F3	RECALL	0.68	0.66	F8	RECALL	0.58	0.57
	SPECIFICITY	0.9	0.89		SPECIFICITY	0.86	0.87
	PRECISION	0.71	0.65		PRECISION	0.57	0.57
	BALANCED ACCURACY	0.79	0.77		BALANCED ACCURACY	0.72	0.72
	F1-SCORE	0.72	0.7		F1-SCORE	0.61	0.62
RANDOM FOREST				SVM			
FOLD	METRICS	TRAINING	TEST	FOLD	METRICS	TRAINING	TEST
F3	RECALL	0.67	0.69	F3	RECALL	0.72	0.69
	SPECIFICITY	0.9	0.91		SPECIFICITY	0.92	0.91
	PRECISION	0.74	0.75		PRECISION	0.77	0.75
	BALANCED ACCURACY	0.79	0.8		BALANCED ACCURACY	0.82	0.8
	F1-SCORE	0.73	0.75		F1-SCORE	0.78	0.76
RIDGE				LASSO			
FOLD	METRICS	TRAINING	TEST	FOLD	METRICS	TRAINING	TEST
F6	RECALL	0.61	0.59	F10	RECALL	0.66	0.63
	SPECIFICITY	0.88	0.88		SPECIFICITY	0.89	0.89
	PRECISION	0.65	0.61		PRECISION	0.66	0.63
	BALANCED ACCURACY	0.75	0.73		BALANCED ACCURACY	0.77	0.76
	F1-SCORE	0.67	0.66		F1-SCORE	0.69	0.68

Considering the rankings, Random Forest and SVM perform the best in terms of balanced accuracy. However, it's important to note that this ranking is based on the provided limited metrics, and the final choice of the best model should consider other factors such as interpretability, computational complexity, and specific requirements of the problem.

## Conclusions

It can be observed that attribute reduction using LDA and PCA had mixed effects on the performance of the classification models. Some models showed improved accuracy after attribute reduction, while others experienced a decrease in accuracy. The Random Forest model achieved the highest testing accuracy of 96.93% before attribute reduction. However, after attribute reduction, its accuracy decreased to 74.91% with LDA and 75.82% with PCA. On the other hand, Logistic Regression showed a slight improvement in testing accuracy after attribute reduction with both LDA (from 83.14% to 75.18%) and PCA (from 83.14% to 79.04%). It is important to note that the choice of attribute reduction technique and the impact on model performance can vary depending on the specific dataset. Further analysis and experimentation are required to determine the most effective approach for attribute reduction and to handle imbalanced data in Medical Data images. The next paper discusses the ensemble model.
